# Eating Vegetables First at Start of Meal and Food Intake among Preschool Children in Japan

**DOI:** 10.3390/nu12061762

**Published:** 2020-06-12

**Authors:** Jiaxi Yang, Yukako Tani, Deirdre K. Tobias, Manami Ochi, Takeo Fujiwara

**Affiliations:** 1 Department of Epidemiology, Harvard T. H. Chan School of Public Health, 677 Huntington Ave, Boston, MA 02215, USA; jiaxiyang@g.harvard.edu; 2 Department of Global Health Promotion, Tokyo Medical and Dental University, 1-5-45 Yushima, Bunkyo-ku, Tokyo 113-8510, Japan; tani.hlth@tmd.ac.jp; 3 Department of Nutrition, Harvard T. H. Chan School of Public Health, 677 Huntington Ave, Boston, MA 02215, USA; dtobias@bwh.harvard.edu; 4 Division of Preventive Medicine, Department of Medicine, Brigham and Women’s Hospital, 900 Commonwealth Avenue, Boston, MA 02215, USA; 5 Department of Health and Welfare Services, National Institute of Public Health, 2-3-6 Minami, Wako-shi, Saitama 351-0197, Japan; ochi.m.aa@niph.go.jp

**Keywords:** dietary habit, vegetable consumption, food intake, preschool children, Japan, nutrition

## Abstract

Eating behavior is an important aspect for dietary quality and long-term health. This study examined associations between eating vegetables first at a meal and food intakes among preschool children in Tokyo, Japan. We used cross-sectional data of 135 preschool children from seven nursery schools in Adachi City, Tokyo, Japan. Caregivers completed a survey on child’s eating behaviors and a diet questionnaire. Linear regression was used to examine frequency of eating vegetables first at a meal and food intakes; percent difference and the corresponding 95% confidence interval (95% CI) were presented. Overall, 25.2% of children reported eating vegetables first at a meal every time, 52.6% sometimes, and 22.2% not often or never. In the multivariate analysis, higher vegetable intake remained significant after adjusting for other covariates (compared with the group of eating vegetables first not often or never, the group reported sometimes: 27%, 95% CI: 0–63%; the group reported every time: 93%, 95% CI: 43–159%). No significant difference in intake by frequency categories of eating vegetables first was observed for other food groups, including fruits, meat, fish, cereals, and sweets. Children eating vegetables first at a meal more was associated with higher total intake of vegetables compared with children who did not eat vegetables first, among Japanese preschool children.

## 1. Introduction

A healthy diet is an essential component for meeting proper nutrition requirement for optimal body growth and body weight during childhood [1,2,3]. A poor diet can be a result of an imbalanced diet by overeating low-nutrient-dense foods such as refined carbohydrates and sweets and failing to consume other foods with high nutrient density, such as fruits, vegetables, and healthy meats [4]. Having a poor diet by consuming an excessive amount of unhealthy food and an insufficient amount of healthy food can lead to both short-term and long-term negative consequences on children development, such as obesity, nutrition deficiency, and insufficient body growth [5,6,7]. In addition to its beneficial role on body growth in childhood, a healthy diet pattern formed in childhood will also benefit long-term health if the pattern is maintained in later life [8,9].

Eating behaviors can reflect and even potentially influence overall dietary quality [10,11]. Particularly, choice of foods consumed during the early phase of a meal with respect to energy intake and intakes of different food groups has been examined in several studies conducted in the United States (U.S.) among people of different age groups. For instance, a randomized cross-over study conducted among adults in the U.S. found that consuming a first course with low-energy-dense salad enhanced satiety and led to reduced energy intake in the subsequent courses [12]. The beneficial role of consuming low-energy-dense foods at the beginning of a meal, particularly vegetables, has also been examined among children. More importantly, studies have revealed that consuming vegetables during the early phase of a meal led to not only reduced meal energy intake but also increased vegetable intake. A study conducted at a daycare center in Pennsylvania by the same research group found that serving low-energy-dense vegetable soup during the early phase of a meal led to reduced intake of energy-dense entrée and increased vegetable consumption at the meal [13]. Another cross-over study examined portion size of vegetables served at the start of meal among preschool children and reported that increasing portion size of vegetables at the start of a meal led to greater vegetable consumption without increasing meal energy intake [14]. Therefore, serving vegetables at the start of a meal and avoiding presence of competing foods that are less healthy may be advocated as an effective strategy to promote higher vegetable intake in preschool children [14].

Lately, there has been a trend of decreasing in vegetable intake and increasing in meat intake among the general Japanese population. The annual survey on citizens’ health and nutrition published by the Ministry of Health, Labor and Welfare in Japan suggested that, compared with 10 years ago, the daily intakes of vegetables and fruits among Japanese had been decreasing (277.4 g/day for vegetables with a drop of 18.4 g/day, 110.3 g/day for fruits with a drop of 22.0 g/day), whereas the daily intake of meat had been increasing (80.7 g/day with an increase of 6.7 g/day) [15]. Since one’s dietary habit is often formed as early as in childhood, identifying children with insufficient vegetable intake and subsequently developing dietary guidelines and interventions that will likely lead to increased vegetable intake and other healthy food intakes should be considered as a useful strategy to address the current diet problem in Japan [9,16].

A typical Japanese meal set usually consists of a staple food (mostly rice), three side dishes, and a soup served all at once [17]. Therefore, the sequence of food consumed can vary from person to person. As suggested by the earlier studies conducted in the U.S. on consuming vegetables during early phase of a meal, evaluating frequency of eating vegetables first at a meal with respect to various food intakes will be useful to determine if early consumption of vegetables at a meal is informative of assessing vegetable consumption and possibly overall dietary quality among Japanese children.

We used data from a cross-sectional study of preschool children in Japan and evaluated the association between frequency of eating vegetables first at a meal and intakes of different food groups, including vegetables, fruits, meat, fish, cereal, and sweets. We hypothesized that frequently consuming vegetables first at a meal would be associated with higher intake of vegetables and other healthier foods.

## 2. Materials and Methods

### 2.1. Study Population

We used data from a cross-sectional study of 135 preschool children from Adachi City, Tokyo, Japan in 2017. The study was initiated as a component of a health promotion campaign known as “Eat Vegetable First at Meals”, which was launched in Adachi City in 2013. Children in 5-year-old class from seven licensed public nursery schools in Adachi City were invited to participate in the study. Teachers at the nursery schools explained to the children’s caregivers about the study and distributed the study questionnaires. The questionnaires included a survey on regular eating behaviors of the participated child and a brief-type diet history questionnaire developed for Japanese preschool children aged 3–6 years (BDHQ3y) [18]. Participants were informed that participation in the study was voluntary and returning the completed questionnaires indicated their consent to participating in the study. The questionnaires were distributed to 165 caregivers, out of which 135 caregivers returned the questionnaires in sealed envelopes via each nursery school (response rate: 81.8%). Use of the data for this study was approved by the Ethics Committee of Tokyo Medical and Dental University (No. M2016-284).

### 2.2. Survey on Dietary Behavior

The dietary behavior survey was filled out by the caregiver of the participated child. It aimed to assess the regular eating and cooking behaviors of both the caregiver and the child. For our study, we were particularly interested in the frequency of eating vegetables first at a meal. In the dietary behavior survey, frequency of eating vegetables first at a meal was assessed in the following question: “how often does your child eat the first bite from vegetables at a given meal?” The caregiver was asked to circle the answer that best applied to his or her child from the following options: “every time”, “sometimes”, “not often”, or “never”. Since there were only 4 children who reported “never” for consuming the first bite from vegetables at a meal, we collapsed the groups of children who reported “not often” or “never” into one group in the analysis.

### 2.3. Survery on Food Intake

Food intakes were assessed using a brief-type diet history questionnaire for Japanese preschool children aged 3–6 years (BDHQ3y), which was developed based on the adult version of a self-administered diet history questionnaire that has been widely used in a range of epidemiologic studies for assessing food intakes in Japanese adults [19]. The caregiver reported the regular food intakes of his or her child during the preceding month by filling out BDHQ3y. The validity of BDHQ3y has been previously tested [18]. Details of BDHQ3y have been described elsewhere [18,20]. Briefly, BDHQ3y is a four-page questionnaire which reflects the typical Japanese dietary pattern, and it includes four sections to assess the food intake frequency: (1) 57 food and nonalcoholic beverage items; (2) daily intakes of rice (the most widely consumed staple food in Japan) and miso soup (widely consumed soup type in Japan); (3) usual cooking methods; and (4) general dietary behaviors. The daily intakes of 66 food items, total energy intake, and nutrient values are then estimated using an ad hoc computer algorithm, which takes into account the age-specific portion size using a specific weighting factor to adjust for the effect of age on the portion size consumed.

We considered the following food groups in our analysis: vegetable, fruit, meat (excluding fish), fish, cereal (including rice, noodles, and bread), and sweets. In the BDHQ3y, vegetable intake was collected based on the consumptions of dark green-leaf vegetables, cabbage, carrots, pumpkins, rooted vegetables, tomatoes, and mushrooms. Fruit intake was collected based on the commonly consumed fruits, except juice and jam made from fruits. Meat intake was collected based on the consumptions of chicken, pork, beef, processed meat, and animal liver. Fish intake was collected based on the intakes from fresh fish, canned fish, dried and salted fish, and food made from fish. Cereal intake was categorized into three sub-groups, and the intake of each group was calculated respectively: rice (including plain white rice, barley, whole grain rice, brown rice, and multigrain rice); noodles (including buckwheat noodles, Japanese wheat noodles, Chinese noodles, fried noodles, instant noodles, and western-style noodles); and bread. Intake on sweets was assessed from the following food sources: western sweets, Japanese sweets, ice cream, chocolate, and other sweet snacks. For each of the food groups, daily intakes of the food items were then summed and a value of total daily intake (g/day) was obtained, respectively. The summed value of the daily intakes from rice, noodles, and bread was reported as the daily intake of cereal. In addition, total energy intake (kcal/day) was also assessed in our study.

For each of the food groups, from the value of daily intake (g/day) estimated based on the nutrient database, we divided the food intake by the total energy intake and then multiplied the value by 1000 to derive the nutrient density (g/1000 kcal per day), so the food intake was represented as a dietary composition (a percentage from daily energy intake) rather than the absolute intake value for each child [4,21]. Nutrient density values were then log-transformed to account for potential non-normality.

### 2.4. Covariates

Information on the child’s date of birth, sex, height, and weight were filled out by the caregiver in the BDHQ3y questionnaire. Information on the following covariates was additionally collected from the dietary behavior survey: number of people in the household, household economic status (in good standing, normal, indigent), parents’ job (self-owned business, full-time, part-time, other), caregiver-rated child’s physical health (good, normal, poor), and child’s physical activity status, which was assessed by the frequency of conducting physical exercise that was longer than 30 min (almost every day, 5–6 times a week, 3–4 times a week, 1–2 times a week, rarely or never). In addition, the caregiver was asked to recall the average frequency of the child’s vegetable consumption (almost at every meal, twice in a day, less than once in a day). We considered the covariates mentioned above as potential confounders for the association between frequency of eating vegetables first at a meal and food intakes and subsequently examined them in the analysis. For any question that was not answered by the caregiver, the missing value was set to the most commonly reported response.

### 2.5. Statistical Analysis

The main exposure of interest was frequency of eating vegetables first at a meal. The exposure was evaluated as a categorical variable with the following category: every time, sometimes, and not often or never. The group of children reported as not often or never eating vegetables first at a meal was set as the reference category. We first examined the association between frequency of eating vegetables first at a meal and other demographic or lifestyle-related covariates by conducting a chi-square test for a categorical covariate and analysis of variance for a continuous covariate. For the univariate analysis, the frequency of eating vegetables first at a meal was included as the only predictor in the univariate model. For the multivariate analysis, covariates with a *p*-value less than 0.05 from the chi-square test or the analysis of variance test were considered as significant and were subsequently adjusted in the multivariate model: age (months), physical health status (good, normal, poor), frequency of consuming vegetables (almost at every meal, twice in a day, less than once in a day). For both univariate and multivariate analyses, we used a linear regression model and examined the association between frequency of eating vegetables first at a meal and intake of the food groups. Since the outcome of food intake was on a logarithmic scale, coefficients and standard errors were back-transformed to the original scale with an interpretation of percent difference in the daily nutrient density for a given group compared to the reference group. All analyses were conducted using STATA version 13 (STATA Statistical Software: Release 13. College Station, TX, USA: StataCorp LP).

## 3. Results

Characteristics of the overall study sample by the reported frequency of eating vegetables first at a meal are summarized in Table 1. Our study included 135 Japanese preschool children with average age of 6.4 years (SD = 0.3 years) and average body mass index (BMI) of 15.5 kg/m^2^ (SD = 1.8 kg/m^2^). With respect to the frequency of eating vegetables first at a meal reported by the caregiver, 34 (25.2%) participants reported “every time”, 71 (52.6%) participants reported “sometimes”, and 30 (22.2%) participants reported “not often or never”. Compared with the other two groups, the group of children reported as eating vegetables first at a meal every time had slightly higher BMI, a greater proportion of parents who owned self-business or had full-time job, better caregiver-rated physical health, more frequent physical activity, as suggested by a lower proportion of children who rarely or never conducted exercise that was longer than 30 min, and more frequent vegetable consumption (Table 1).

Daily intakes of the major food groups (g/1000 kcal, except for total energy intake) with respect to the frequency of eating vegetables first at a meal are summarized in Table 2. As Table 2 suggests, we observed higher total vegetable intake independent of total energy intake in the groups of children reported as more frequently eating vegetables first at a meal (every time: 147.8 g/1000 kcal, sometimes: 88.7 g/1000 kcal, not often or never: 68.0 g/1000 kcal). Higher intakes of fruits and fish and lower intakes of cereal and sweets were also observed in the group of eating vegetables first every time compared with the other two groups with the lower frequency. We did not observe a difference in total energy intake across the three groups (Table 2).

We present our main analysis results in Table 3. In the univariate analysis, we observed a significant association between frequently eating vegetables first at a meal and higher total vegetable intake (Table 3). Compared with the group of eating vegetables first at a meal not often or never, we observed 46% (95% CI: 14–88%) higher vegetable intake in the “sometimes” group and 139% (95% CI: 79–219%) higher vegetable intake in the “every time” group. In addition, significantly higher intakes of fruits and fish and lower intake of bread were also observed in the group of eating vegetables first at a meal every time compared with the reference group (Table 3).

After adjusting for the relevant covariates (age, physical health status, frequency of consuming vegetables) in the multivariate model, the association between frequently eating vegetables first at a meal and higher intake of vegetables was slightly attenuated, but it still remained statistically significant (Table 3). Compared with the group of children reported as eating vegetables first at a meal not often or never, the “sometimes” group had 27% (95% CI: 0–63%) higher vegetable intake, and the “every time” group had 93% (95% CI: 43–159%) higher vegetable intake. We did not observe significant associations between frequency of eating vegetables first at a meal and food intake for the remaining food groups that we examined, including fruits, meat, fish, cereal, and sweets (Table 3).

## 4. Discussion

In our analysis of 135 Japanese preschool children, we found that frequently eating vegetables first at a meal was associated with higher intake of vegetables, and suggestively higher intakes of fruits and fish and lower intake of bread, independent of energy intake. To our knowledge, this is the first study examining frequency of eating vegetables first at a meal and its association with intake of various food groups among Japanese preschool children. The multi-dish style in the Japanese meal culture allowed us to closely examine the role of eating vegetables first at a meal on the intakes of commonly consumed foods among Japanese preschool children.

Our results were consistent with the previous study findings on consuming vegetables during early phase of a meal and greater vegetable consumption. A cross-over study conducted among preschool children in the U.S. found that doubling the portion size of vegetables as the first course led to a subsequent 47% increase in vegetable consumption at a given meal [14]. The same research group conducted other studies examining the role of serving vegetable dishes in the early phase of a meal. They reported similar findings that consuming a vegetable dish early led to increased meal vegetable intake and decreased meal energy intake [12,13]. Based on these study findings, placing vegetable dishes earlier during the course of a meal can be advocated as a strategy to encourage vegetable intake among children who have insufficient vegetable consumptions. Indeed, serving-vegetable-first has been demonstrated as an effective way to increase vegetable consumption among school children in other settings [22].

In addition to the significant association with higher vegetable intake, we also observed that frequently eating vegetables first at a meal was associated with suggestively higher intakes of fruits and fish, and it was not associated with higher intakes of the food groups that were considered less healthy, such as bread and sweets. In fact, compared with the group of children reported as not often or never eating vegetables first at a meal, the group of children eating vegetables first every time had suggestive lower intakes in bread and sweets (Table 2 and Table 3). Further, eating vegetables first more frequently at a given meal did not seem to be associated with increased meal energy intake, which was also consistent with the previous study findings (Table 2) [14]. Therefore, it may be implied that frequently eating vegetables first at a meal was not associated with higher intake of unhealthy food or higher intake of energy. Considering the healthy benefits of eating vegetables, fruits, and fish and current dietary guidelines on limiting intake of refined carbohydrates, our results suggested the possibility of using frequency of eating vegetables first at a meal as a useful tool to assess the overall dietary quality among Japanese preschool children [16,23,24,25,26,27,28,29,30,31].

Our study provided preliminary evidence that assessing frequency of eating vegetables first at a meal might serve as a convenient and useful method for the policymakers to identify the population of children with generally low vegetable consumption and possibly suboptimal diet quality, and to subsequently develop community interventions or guidelines to improve their diet. Findings from our study also provide useful insights for future interventional studies to further pursue this area of research in order to draw causal conclusions on frequency of eating vegetables first at a meal and increasing total vegetable intake among preschool children in Japan.

There are some limitations in our study. First, with a small sample size (*n* = 135), the statistical power of our study was limited. Therefore, the null associations observed in some food groups may be interpreted as either no association or a possible association but underpowered. In addition, since we collapsed the groups of “not often” and “never” into one group due to the limited sample size, we were unable to separately examine the food intakes for those two groups. Furthermore, a small sample size may limit the generalizability of our results. Therefore, future studies with greater sample size and sufficient statistical power should be conducted to address such limitations. Second, given the cross-sectional nature of the study, our results can only be interpreted as findings of associations. Therefore, we cannot make the causal interpretation that eating vegetables first at a meal will lead to higher vegetable intake. However, our results still suggest that frequently eating vegetables first at the start of a meal is informative of higher total vegetable consumption among Japanese preschool children. Lastly, similar to other nutritional studies, diet was likely to be measured with errors, as the validity and reliability of BDHQ3y filled out by the caregiver may not be high enough to accurately capture the regular dietary pattern of the Japanese preschool children. With respect to the dietary behaviors survey, since it was structured as questions with a reasonable number of choices, misclassification was likely to be low.

## 5. Conclusions

In conclusion, our study on 135 Japanese preschool children suggested that compared with children who did not eat vegetables first, eating vegetables first at a meal more was associated with a higher total vegetable intake. Larger-scale studies with a geographically diverse population of preschool children should be conducted to further confirm our findings. Future intervention studies or randomized trials are warranted to further examine the causal role of eating vegetables first at a meal on increasing healthy foods consumptions among Japanese preschool children.

## Figures and Tables

**Table 1:** Population characteristics and characteristics by frequency of eating vegetables first at a meal.

		Frequency of Eating Vegetables First at a Meal			
	Total (*n* = 135)	Every Time (*n* = 34, 25.2%)	Sometimes (*n* = 71, 52.6%)	Not Often or Never (*n* = 30, 22.2%)	*p*-Value ^1^
	Mean (SD) ^2^				
Age (years) ^3^	6.4 (0.3)	6.3 (0.3)	6.4 (0.3)	6.5 (0.3)	0.03
BMI (kg/m^2^) ^4^	15.5 (1.8)	15.9 (1.7)	15.4 (1.6)	15.3 (2.2)	0.42
Family size ^5^	3	3	3	3	0.08
	Count (percent)				
Male	67 (49.6%)	15 (44.1%)	34 (47.9%)	18 (60.0%)	0.41
Economic status					0.89
In good standing	13 (9.6%)	3 (8.8%)	6 (8.5%)	4 (13.3%)	
Normal	93 (68.9%)	24 (70.6%)	48 (67.6%)	21 (70.0%)	
Indigent	29 (21.5%)	7 (20.6%)	17 (23.9%)	5 (16.6%)	
Job of mother					0.38
Self-owned business	10 (7.4%)	3 (8,8%)	4 (5.6%)	3 (10.0%)	
Full-time	63 (46.7%)	20 (58.8%)	32 (45.1%)	11 (36.7%)	
Part-time	53 (39.3%)	10 (29.4%)	28 (39.4%)	15 (50.0%)	
Other	9 (6.7%)	1 (2.9%)	7 (9.9%)	1 (3.3%)	
Job of father					0.64
Self-business	15 (11.1%)	3 (8.8%)	6 (8.5%)	6 (20.0%)	
Full-time	94 (69.6%)	25 (73.5%)	52 (73.2%)	17 (56.7%)	
Part-time	6 (4.4%)	1 (2.9%)	3 (4.2%)	2 (6.7%)	
Other	20 (14.8%)	5 (14.7%)	10 (14.1%)	5 (16.7%)	
Physical health status					0.01
Good	106 (78.5%)	33 (97.1%)	50 (70.4%)	23 (76.7%)	
Normal	21 (15.6%)	1 (2.9%)	17 (23.9%)	3 (10.0%)	
Poor	8 (5.9%)	0	4 (5.6%)	4 (13.3%)	
Frequency of conducting physical activity (longer than 30 min)					0.69
Almost every day	17 (12.6%)	5 (14.7%)	6 (8.5%)	6 (20.0%)	
5–6 times a week	9 (6.7%)	3 (8.8%)	5 (7.0%)	1 (3.3%)	
3–4 times a week	24 (17.8%)	8 (23.5%)	13 (18.3%)	3 (10.0%)	
1–2 times a week	66 (48.9%)	14 (41.2%)	36 (50.7%)	16 (53.3%)	
Rarely or never	19 (14.1%)	4 (11.8%)	11 (15.5%)	4 (13.3%)	
Frequency of consuming vegetables					<0.01
Almost at every meal	57 (42.2%)	20 (58.8%)	29 (40.9%)	8 (26.7%)	
Twice in a day	60 (44.4%)	13 (28.2%)	35 (49.3%)	12 (40.0%)	
Less than once in a day	18 (13.3%)	1 (2.9%)	7 (9.8%)	10 (33.3%)	

^1^*p*-value from chi-square test for categorical covariate and analysis of variance for continuous covariate is presented. ^2^ SD: standard deviation. ^3^ Age is presented in years by dividing age in months by 12. ^4^ BMI: body mass index. ^5^ Median is presented for the number of people in the household.

**Table 2:** Summary of major food group intakes by frequency of eating vegetables first at a meal.

		Frequency of Eating Vegetables First at a Meal		
Daily Food Intake	Total (*n* = 135)	Every Time (*n* = 34, 25.2%)	Sometimes (*n* = 71, 52.6%)	Not Often or Never (*n* = 30, 22.2%)
	Mean (SD)			
Total energy intake (kcal)	1427.1 (471.7)	1442.6 (408.1)	1405.51 (445.9)	1460.5 (596.9)
Vegetables (g/1000 kcal)	99.0 (68.5)	147.8 (88.5)	88.7 (50.1)	68.0 (51.7)
Fruits (g/1000 kcal)	40.9 (29.4)	56.6 (31.1)	33.8 (24.5)	39.9 (32.0)
Meat excluding fish (g/1000 kcal)	32.3 (14.3)	33.7 (12.8)	33.3 (13.9)	31.2 (16.9)
Fish (g/1000 kcal)	31.4 (16.0)	34.6 (15.1)	30.9 (15.8)	28.6 (17.4)
Cereal (g/1000 kcal) ^1^	240.7 (63.4)	230.7 (59.1)	241.9 (56.7)	549.5 (81.4)
Rice	189.3 (66.1)	188.4 (59.6)	188.3 (59.9)	192.7 (86.5)
Noodles	32.5 (20.1)	27.5 (14.6)	33.56 (18.7)	35.4 (27.2)
Bread	19.0 (13.1)	14.7 (11.3)	20.0 (13.8)	21.4 (12.5)
Sweets (g/1000kcal)	34.8 (21.5)	29.1 (17.5)	36.8 (21.4)	36.4 (25.1)

^1^ Cereal intake was calculated as the summed value of intakes from rice, noodles, and bread.

**Table 3:** Results on association between frequency of eating vegetables first at a meal and types of food intake ^1^.

Daily Food Intake ^1^	Frequency of Eating Vegetables First at a Meal	Univariate Model ^2^	Multivariate Model ^3^
		Percent Difference (95% CI)	
Vegetables	Not often or never	Reference	
	Sometimes	46% (14%, 88%)	27% (0%, 63%)
	Every time	139% (79%, 219%)	93% (43%, 159%)
Fruits	Not often or never	Reference	
	Sometimes	−9% (−34%, 26%)	−21% (−43%, 11%)
	Every time	51% (4%, 118%)	23% (−17%, 82%)
Meat excluding fish	Not often or never	Reference	
	Sometimes	12% (−8%, 35%)	8% (−12%, 32%)
	Every time	15% (−8%, 43%)	9% (−15%, 39%)
Fish	Not often or never	Reference	
	Sometimes	14% (−12%, 49%)	15% (−13%, 52%)
	Every time	36% (1%, 86%)	30% (−7%, 82%)
Cereal ^4^	Not often or never	Reference	
	Sometimes	0% (−12%, 14%)	3% (−10%, 19%)
	Every time	−5% (−18%, 10%)	0% (−16%, 17%)
Rice	Not often or never	Reference	
	Sometimes	4% (−13%, 25%)	3% (−16%, 25%)
	Every time	4% (−15%, 28%)	5% (−17%, 33%)
Noodles	Not often or never	Reference	
	Sometimes	−5% (−26%, 23%)	3% (−22%, 35%)
	Every time	−20% (−40%, 8%)	−15% (−39%, 19%)
Bread	Not often or never	Reference	
	Sometimes	−13% (−38%, 21%)	3% (−28%, 48%)
	Every time	−40% (−59%, −11%)	−31% (−55%, 5%)
Sweets	Not often or never	Reference	
	Sometimes	8% (−18%, 43%)	3% (−24%, 39%)
	Every time	−13% (−37%, 20%)	−15% (−41%, 22%)

^1^ Food intake was calculated as nutrient density (g/1000 kcal per day) for each food type on the natural log scale (nutrient density was calculated by dividing reported daily food intake (g/day) by total energy intake and then multiplying by 1000). ^2^ Frequency of eating vegetables first at a meal was included in the univariate model. ^3^ Multivariate model was adjusted for age (months), physical health status (good, normal, poor), and frequency of consuming vegetables (almost every meal, twice in a day, less than once in a day). ^4^ Cereal intakes were calculated as the summed value of intakes from rice, noodles, and bread.

## References

[1] Emmett P.M., Jones L.R. (2015). Diet, growth, and obesity development throughout childhood in the Avon Longitudinal Study of Parents and Children. Nutr. Rev..

[2] Wu X.Y., Zhuang L.H., Li W., Guo H.W., Zhang J.H., Zhao Y.K., Hu J.W., Gao Q.Q., Luo S., Ohinmaa A. (2019). The influence of diet quality and dietary behavior on health-related quality of life in the general population of children and adolescents: A systematic review and meta-analysis. Qual. Life Res..

[3] Alimujiang A., Colditz G.A., Gardner J.D., Park Y., Berkey C.S., Sutcliffe S. (2018). Childhood diet and growth in boys in relation to timing of puberty and adult height: The Longitudinal Studies of Child Health and Development. Cancer Causes Control..

[4] Willett W.C., Howe G.R., Kushi L.H. (1997). Adjustment for total energy intake in epidemiologic studies. Am. J. Clin. Nutr..

[5] Dehghan-Kooshkghazi M., Akhtar-Danesh N., Merchant A.T. (2005). Childhood obesity, prevalence and prevention. Nutr. J..

[6] Rogol A.D., A Clark P., Roemmich J.N. (2000). Growth and pubertal development in children and adolescents: Effects of diet and physical activity. Am. J. Clin. Nutr..

[7] Scaglioni S., Agostoni C., De Notaris R., Radaelli G., Radice N., Valenti M., Giovannini M., Riva E. (2000). Early macronutrient intake and overweight at five years of age. Int. J. Obes..

[8] Kelder S.H., Perry C.L., I Klepp K., Lytle L.L. (1994). Longitudinal tracking of adolescent smoking, physical activity, and food choice behaviors. Am. J. Public Health.

[9] Reilly J.J. (2008). Physical activity, sedentary behaviour and energy balance in the preschool child: Opportunities for early obesity prevention. Proc. Nutr. Soc..

[10] Patrick H., Nicklas T.A. (2005). A review of family and social determinants of children’s eating patterns and diet quality. J. Am. Coll. Nutr..

[11] Bellisle F. (2014). Meals and snacking, diet quality and energy balance. Physiol. Behav..

[12] Rolls B.J., Roe L.S., Meengs J.S. (2004). Salad and satiety: Energy density and portion size of a first-course salad affect energy intake at lunch. J. Am. Diet. Assoc..

[13] Spill M.K., Birch L.L., Roe L.S., Rolls B.J. (2011). Serving large portions of vegetable soup at the start of a meal affected children’s energy and vegetable intake. Appetite.

[14] Spill M.K., Birch L.L., Roe L.S., Rolls B.J. (2010). Eating vegetables first: The use of portion size to increase vegetable intake in preschool children. Am. J. Clin. Nutr..

[15] Ministry of Health, Labour and Welfare (Japan) (2011). Japan National Health and Nutrition Survey. https://www.mhlw.go.jp/bunya/kenkou/eiyou/h23-houkoku.html.

[16] Mikkila V., Räsänen L., Raitakari O., Pietinen P., Viikari J. (2005). Consistent dietary patterns identified from childhood to adulthood: The Cardiovascular Risk in Young Finns Study. Br. J. Nutr..

[17] Ministry of Agriculture, Forestry and Fisheries (Japan) WASHOKU. http://www.maff.go.jp/j/keikaku/syokubunka/culture/pdf/guide_all.pdf.

[18] Asakura K., Haga M., Sasaki S. (2015). Relative Validity and Reproducibility of a Brief-Type Self-Administered Diet History Questionnaire for Japanese Children Aged 3–6 Years: Application of a Questionnaire Established for Adults in Preschool Children. J. Epidemiol..

[19] Kobayashi S., Murakami K., Sasaki S., Okubo H., Hirota N., Notsu A., Fukui M., Date C. (2011). Comparison of relative validity of food group intakes estimated by comprehensive and brief-type self-administered diet history questionnaires against 16 d dietary records in Japanese adults. Public Health Nutr..

[20] Kano M., Tani Y., Ochi M., Sudo N., Fujiwara T. (2019). Association Between Caregiver’s Perception of “Good” Dietary Habits and Food Group Intake Among Preschool Children in Tokyo, Japan. Front. Pediatr..

[21] Willett W. (2012). Nutritional Epidemiology.

[22] Elsbernd S., Reicks M., Mann T., Redden J.P., Mykerezi E., Vickers Z. (2016). Serving vegetables first: A strategy to increase vegetable consumption in elementary school cafeterias. Appetite.

[23] Slavin J.L., Lloyd B. (2012). Health Benefits of Fruits and Vegetables1. Adv. Nutr..

[24] Aune D., Giovannucci E., Boffetta P., Fadnes L.T., Keum N., Norat T., Greenwood D.C., Riboli E., Vatten L.J., Tonstad S. (2017). Fruit and vegetable intake and the risk of cardiovascular disease, total cancer and all-cause mortality-a systematic review and dose-response meta-analysis of prospective studies. Int. J. Epidemiology.

[25] Yip C.S.C., Chan W., Fielding R. (2019). The Associations of Fruit and Vegetable Intakes with Burden of Diseases: A Systematic Review of Meta-Analyses. J. Acad. Nutr. Diet..

[26] DeSalvo K.B., Olson R., Casavale K.O. (2016). Dietary Guidelines for Americans. JAMA.

[27] Frost L., Vestergaard P. (2005). n-3 Fatty acids consumed from fish and risk of atrial fibrillation or flutter: The Danish Diet, Cancer, and Health Study. Am. J. Clin. Nutr..

[28] Jacobs D.R., Marquart L., Slavin J., Kushi L.H. (1998). Whole-grain intake and cancer: An expanded review and meta-analysis. Nutr. Cancer.

[29] Gaesser G.A. (2007). Carbohydrate Quantity and Quality in Relation to Body Mass Index. J. Am. Diet. Assoc..

[30] Steyn N.P., Mann J., Bennett P.H., Temple N., Zimmet P., Tuomilehto J., Lindstrom J., Louheranta A. (2004). Diet, nutrition and the prevention of type 2 diabetes. Public Heal. Nutr..

[31] Burke J.D. (2015). Dietary Guidelines for Americans. Nutr. Today.

